# Modulating the inflammatory properties of activated microglia with Docosahexaenoic acid and Aspirin

**DOI:** 10.1186/1476-511X-12-16

**Published:** 2013-02-11

**Authors:** Lauren K Pettit, Christopher Varsanyi, James Tadros, Evros Vassiliou

**Affiliations:** 1 Kean University, 1000 Morris Avenue, Union, NJ, 07083, USA; 2 Colfax Oncology Center, 680 Broadway, Paterson, NJ, 07514, USA

**Keywords:** Microglia, Docosahexaenoic acid, Aspirin, Inflammation, Cytokines, Nitric oxide, Glutathione

## Abstract

**Background:**

Microglia are considered the “resident macrophages” of the brain. When in their resting state, microglia perform routine maintenance and immune surveillance. Once activated, either by injury or an immune stimulus, microglia secrete a variety of pro-inflammatory molecules, such as Nitric Oxide, superoxide, and inflammatory cytokines. Up-regulation of pro-inflammatory molecules is transient, and does not cause neurodegeneration. However, if up-regulation lasts for an extended period of time, neurodegeneration ensues.

Many neurodegenerative diseases are characterized by chronic inflammation due to microglial activation. Non-Steroidal Anti-Inflammatory Drugs (NSAIDs) have been proposed as possible preventative treatments for neurodegenerative diseases, due to their anti-inflammatory properties. Docosahexaenoic Acid (DHA) is an omega-3 polyunsaturated fatty acid (PUFA) that has potent anti-inflammatory properties.This research work sought to elucidate whether microglial activation can be modulated by combining Aspirin, a classical NSAID, with Docosahexaenoic Acid, a natural anti-inflammatory agent. The combined ability of Aspirin and DHA to modulate microglial activation was determined in the context of pro-inflammatory cytokines, Nitric Oxide levels, as well as total Glutathione levels.

**Results:**

Docosahexaenoic Acid increased total Glutathione levels in microglia cells and enhanced their anti-oxidative capacity. It reduced production of the pro-inflammatory cytokines TNF-α and IL-6 induced through TLR-3 and TLR-4 activation. Furthermore, it reduced production of Nitric Oxide. Aspirin showed similar anti-inflammatory effects with respect to TNF-α during TLR-3 and TLR-7 stimulation. Aspirin did not show any redection in terms of Nitric Oxide production. Combination of Aspirin and Docosahexaenoic Acid showed augmentation in total Glutathione production during TLR-7 stimulation as well as a reduction in IL-6, TNF-α and Nitric Oxide.

**Conclusions:**

Collectively, these findings highlight the combination of Docosahexaenoic Acid and Aspirin as a possible measure against inflammation of the nervous system, thus leading to protection against neurodegenerative diseases with an inflammatory etiology.

## Background

Neurodegeneration is defined as the loss of the structure and function of neurons [1]. Neurodegeneration due to microglial activation and inflammation is seen in many Central Nervous System (CNS) pathologies, especially neurodegenerative diseases (ND) [2,3]. NDs that involve neurodegeneration include Alzheimer’s disease (AD), Amyotrophic Lateral Sclerosis (ALS), Multiple Sclerosis (MS), and Parkinson’s disease (PD) [1,4]. Although these diseases are characterized by neurodegeneration, they differ in the area of the brain that is affected, leading to the different pathologies that exist for each type of ND [1]. In Alzheimer’s disease, chronic inflammation causes neuronal cell death in the areas of the hippocampus and frontal cortex [4]. Inflammation destroys motor neurons in the spinal cord, brain stem, and cortex in Amyotrophic Lateral Sclerosis [4,5]. In Parkinson’s disease, chronic inflammation causes the loss of dopaminergic receptors in the substantia nigra [4]. Lastly, Multiple Sclerosis is an autoimmune disorder where inflammatory cells attack the myelin sheath that surrounds the axons of neurons [4].

The brain is separated from the periphery by the blood brain barrier, which allows the brain to be immuno-priviledged [4]. Inflammation is an activated immune state and is considered a normal self-defense mechanism that is implemented by the body in order to fight pathogens [4]. Inflammation in the body recruits immune cells to the area that is under attack, which will then clear the system of the antigen [4]. When inflammation occurs in the CNS, microglia are recruited to the affected area [4]. Microglia are considered the “resident macrophage” of the brain [2,4]. When in their resting state, microglia perform routine maintenance and immune surveillance [4,5]. Once activated, either by injury or an immune stimulus, microglia secrete a variety of pro-inflammatory molecules, such as Nitric Oxide (NO), superoxide, inflammatory cytokines, reactive oxygen species (ROS), and glutamate [2-6]. Activated microglia also express inducible Nitric Oxide synthase (iNOS), as well as cyclo-oxygenase-2 (COX-2), which cause the production and subsequent release of NO and pro-inflammatory cytokines [7]. Neuronal cell death will not occur if the inflammatory response is transient [4]. However, if a prolonged inflammatory response occurs, chronic inflammation, neurodegeneration and neuronal cell death will also occur [2,4]. Neuronal cell death leads to reactive microgliosis, the activation of microglia as a result of neuronal death [2]. Reactive microgliosis is toxic to surrounding neurons and results in continued microglial activation, inflammation, and neuronal death [2].

Microglia can be activated in a number of ways, including injury or immunological stimuli [3,4]. Microglial activation, due to immunological stimuli, occurs through a Toll-Like Receptor (TLR) pathways [8]. TLR pathways are considered the first line of defense against viral and bacterial pathogens [8]. Toll-like receptors are a family of nine receptors (TLR1-9) that are found on the cell’s plasma membrane and on the surface of endosomal vesicles, which specifically recognize conserved pathogen-associated molecular patterns (PAMPs) that recognize a variety of pathogens (bacteria, viruses, parasites, yeast and fungi) [8]. Microglial expression of TLRs is undetectable when in their resting state [8]. However, once activated, microglia rapidly express a variety of toll-like receptors (TLR1-9) at differing intesities [8]. It should be noted that over stimulation of TLRs can result in chronic inflammation, leading to many inflammatory diseases [8].

Microglial TLRs can be activated by exogenous and endogenous TLR ligands, including Lipopolysaccharide (LPS) and Polyinosinic-Polycytidylic acid (Poly I:C) [8]. LPS, an endotoxin found in gram-negative bacteria activates TLR-4 receptors expressed on microglia [2,8]. Poly I:C activates TLR-3 receptors on microglia by mimicking the viral double stranded RNA observed during viral replication [8]. When microglial TLRs are stimulated by LPS or Poly I:C, signaling occurs and causing the production and subsequent secretion of inflammatory molecules, reactive oxygen species, and glutamate [8]. These molecules are neurotoxic and cause neurodegeneration [8]. The extent of neurodegeneration that occurs depends on the intensity and length of microglial activation [9].

Cytokines are secreted by immune cells under a variety of conditions [4]. Cytokines regulate inflammatory processes and are also key regulators of ND pathologies [4]. As the brain ages, the blood–brain barrier becomes compromised, leading to an increase in the synthesis of pro-inflammatory cytokines such as IL-6, TNF-α and IL-1β [4]. This causes continued microglial activation and neuro-inflammation [4]. IL-6, TNF-α and IL-1β induce expression of the cyclo-oxygenase 2 (COX-2) enzyme. Over expression of COX-2 has been shown to be involved in neuronal apoptosis [4]. TNF-α and IL-1β have also been shown to influence synaptic transmission *in vitro*[10]. TNF-α is also thought to cause neurodegeneration by silencing cell survival signals and activating caspase-dependent pathways [11]. TNF-α causes glutamate release by activated microglia, leading to excitoneurotoxicity and neuronal damage [11]. Excessive IL-6 and IL-1β have been identified as being neurotoxic [10]. However, it is not known if IL-6 and IL-1β are directly causing neurotoxicity, or it is mediated by other molecules such as ROS or glutamate [10]. In regards to specific neurodegenerative diseases, brains affected by Alzheimer’s disease have increased levels of pro-inflammatory cytokines [4]. Pro-inflammatory cytokines have also been suspected of being able to determine the extent of neurodegeneration that is seen in Multiple Sclerosis [10].

Excessive NO production is associated with both acute and chronic inflammation [12]. Neurons are very susceptible to Nitric Oxide-induced death and very low concentrations can cause extensive neuronal damage and death [3,13]. Recent studies suggest that activated microglia kill co-cultured neurons through a NO and ROS-mediated mechanism [3]. Glutamate-mediated excitotoxicity has also been implicated in causing neuronal injury and death [5]. The following mechanism for Nitric Oxide and glutamate-mediated neuronal cell death has been proposed: NO is released by activated microglia and inhibits neuronal respiration, causing glutamate to be released by neurons [3]. Glutamate then binds to NMDA receptors present on neuronal cells, causing an extreme calcium influx, and ultimately neuronal cell death [3].

ROS and oxidative stress have been implicated in neurodegenerative diseases such as Alzheimer’s disease, Parkinson’s disease, and Amyotrophic Lateral Sclerosis [4]. In Alzheimer’s disease, plaque formation and accumulation lead to an inflammatory response that causes the production of ROS, and cause oxidative damage to surrounding neurons [4]. Plaque accumulation also causes oxidative damage, which leads to mitochondrial dysfunction [4]. Together, mitochondrial dysfunction and oxidative damage can also cause amyloid aggregation and tau polymerization [14]. In Parkinson’s disease, increases in the generation of reactive oxygen species, as well as lipid peroxidation, are seen in the substantia nigra [4,14]. Lastly, the pathogenesis of familial and sporadic Amyotrophic Lateral Sclerosis has been shown to involve ROS and oxidative stress [4].

Recent studies suggest that Docosahexaenoic Acid (DHA), a 22-carbon, long-chain polyunsaturated fatty acid (LC-PUFA), could have beneficial effects in brain diseases [15]. The combination of aspirin and DHA has been shown to generate a number of anti-inflammatory species [16]. DHA is a major fatty acid that makes up about 12-16% of total fatty acids in the grey matter of the brain [17]. DHA is important in proper brain development, and plays a role in maintaining a homeostatic environment in the CNS [15,18,19]. DHA has been shown to modulate important neurochemical processes, synaptic plasticity, memory formation, neuroprotection, gene expression, and intracellular calcium concentrations [15,19]. DHA promotes neural stem cell differentiation into neurons, as well as neurogenesis [15]. DHA and its derivatives, whether made by peroxidation or enzymatic processing, all have potent anti-inflammatory properties in both acute and chronic ND [15]. DHA has been shown to reduce the number of activated microglia and reduce pro-inflammatory molecule production [15].

DHA is known for its anti-inflammatory properties and has been shown to reduce pro-inflammatory cytokine production in microglia [7,15]. The NF-κB signaling pathway is important for mediating the expression of genes such as COX-2 and iNOS, which encode pro-inflammatory molecules, such as Nitric Oxide and prostaglandins [7,12,20]. DHA inhibits pro-inflammatory cytokine production by preventing NK-κB translocation to the nucleus [12]. This causes a decrease in the transcription of pro-inflammatory genes (COX-2 and iNOS) [12]. Ultimately, pro-inflammatory cytokines and Nitric Oxide production is reduced [12].

DHA also causes an increased level of intracellular Glutathione (GSH), a potent antioxidant molecule that is found in the brain at high concentrations [21]. High levels of GSH have been shown to be important for suppressing NF-κB activation [12]. Glutathione is also a cofactor for Glutathione Peroxidase (GPx), an enzyme that converts hydrogen peroxide to water [12]. High expression of GPx has been shown to inhibit the degredation of IκB, as well as inhibit the activation of NF-κB [12]. Increased levels of GSH, caused by DHA, enhance the activity of GPx, which further inhibits the transcription and translation of pro-inflammatory molecules through NF-κB signaling [12]. DHA also increases the activity of Glutathione Reductase (GR), an enzyme that is important in maintaining the anti-oxidative capacity of cells by a GSH-based mechanism [12,17]. In turn, the occurrence of up-regulated GPx and GR in the brain causes an increase in GSH, and an enhancement of the anti-oxidative defense mechanism employed by the brain [12].

Nitric Oxide mediates inflammatory processes [13]. Recently, DHA has been shown to reduce iNOS expression and NO production in microglia [15]. DHA has also been shown to inhibit iNOS expression and NO production in murine macrophages [12]. DHA also down-regulates the expression of genes involved in ROS production [22]. It is thought that this reduction occurs through up-regulation of the anti-oxidative capacity of the macrophages by enhancing a Glutathione-mediated anti-oxidative mechanism [12].

Dietary intake is the main source of DHA in humans and when adequate, it offers visual, neurological and cardiovascular health benefits [12-15,17,18]. Decreased DHA intake can lead to oxidative damage, and has been shown to cause cognitive insufficiencies and impaired vision [4,18]. Reduced DHA intake in adults has been shown to contribute to age-related cognitive deficiencies, as well as neuronal dysfunction [15]. Low DHA in the blood is hypothesized to be an important risk factor in developing Alzheimer’s disease [17,23].

Increased dietary intake has been shown to significantly alter DHA levels in the brain [17]. This suggests that DHA supplementation could be used to directly influence brain function [17]. Clinical studies suggest dietary DHA supplementation can alter the risk of developing Alzheimer’s disease [17]. Participants with the highest level of DHA in their blood also had a decreased risk of developing dementia [17]. Moderate increases of DHA in the daily diet have been shown to reduce the risk of developing Alzheimer’s disease by 60% [24]. It should be noted that elderly people who eat fish and seafood enriched with omega-3 PUFAs (i.e.: DHA) at least once a week have a decreased risk of developing dementia and Alzheimer’s disease [25]. The aforementioned data suggests that DHA could be an effective therapy for preventing Alzheimer’s disease, as well as other neurodegenerative diseases [25].

Non-steroidal anti-inflammatory drugs (NSAIDs) have been proposed as a possible preventative treatment for NDs [4]. NSAIDs, as their name describes, have anti-inflammatory properties and can be selective for cyclo-oxygenase (COX) -1, COX-2, or both [4]. NSAIDs inhibit the production of Nitric Oxide, as well as pro-inflammatory cytokine production [26] NSAIDs that specifically target COX-2 have been shown to reduce microglial activation, block the production of pro-inflammatory cytokines, and reduce the risk of Alzheimer’s disease [4]. NSAIDs have also been shown to block the production and accumulation of degenerative proteins, thereby reducing the risk of Alzheimer’s disease [4]. NSAIDs that target COX-2 have been shown to improve cognitive and motor functions in mice [4,23]. Epidemiological studies have shown an inverse relationship between NSAID intake and the development of Alzheimer’s and Parkinson’s diseases [23,27]. NSAIDs are also proposed to have an impact on the inflammatory component of Multiple Sclerosis and Amyotrophic Lateral Sclerosis [17]. Ibuprofen, a NSAID that is non-selective in terms of COX-1 and COX2, has been proposed through epidemiological studies as a possible preventative treatment for Alzheimer’s disease [4]. However, Ibuprofen has concerning side effects that have prevented it from being used in clinical trials for Alzheimer’s prevention [4].

COX-2 in neurons, neurodegeneration caused by excitotoxicity, as well as neurodegernation caused by microglia, are proposed as the main targets of NSAIDs [23]. With this information, classical NSAIDs are logically an attractive option for delaying the onset and slowing the progression of neurodegenerative diseases [28]. Combination therapies of different types of anti-inflammatory agents are also proposed as a preventative therapy for neurodegenerative diseases because they can work through different mechanisms [28].

This research project sought to elucidate whether microglial activation can be modulated by combining Aspirin, a classical NSAID, with Docosahexaenoic Acid, a naturally occurring anti-inflammatory agent. The combined ability of Aspirin and DHA to modulate activated microglia was determined in the context of pro-inflammatory cytokines, Nitric Oxide levels, Reactive Oxygen Species, as well as total Glutathione levels.

## Results and discussion

### Neither DHA pretreatment nor aspirin treatment cause toxicity in activated microglia

**Figure 1:**
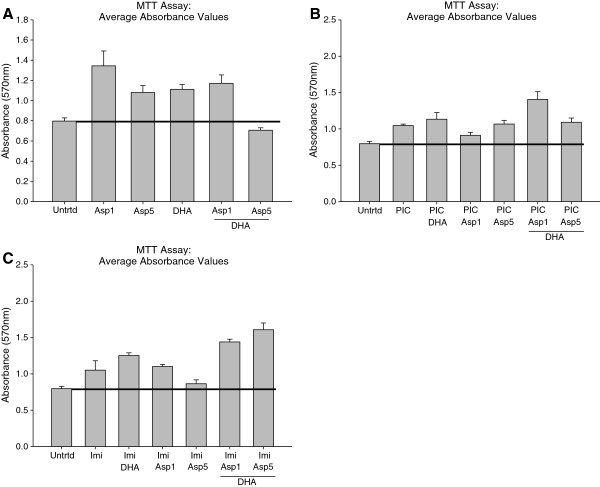
**A. 50,000 EOC20 Microglial Cells were plated in a 96 well plate containing Complete Medium plus 20% LADMAC Conditioned Medium.** Cells were stimulated with 50μM DHA for 24 hours. After 24 hours, cells were stimulated with either Aspirin at 1mM or Aspirin at 5mM. At 24 hours post TLR stimulation and/or Aspirin treatment, the experimental plate was assayed for necrosis using a Non-Radioactive Cell Proliferation kit (MTT Assay). Untrtd = Untreated; Asp1 = Aspirin (1mM); Asp5 = Aspirin (5mM); DHA = Docosahexaenoic Acid (50μM). * = p<0.05; ** = p<0.005. **B**. 50,000 EOC20 Microglia were plated in a 96 well plate containing Complete Medium plus 20% LADMAC Conditioned Medium. Cells were stimulated with 50μM DHA for 24 hours. After 24 hours, cells were stimulated with Poly I:C, Aspirin at 1mM, Aspirin at 5mM, or a combination of Poly I:C and Aspirin. At 24 hours post Poly I:C stimulation and/or Aspirin treatment, the experimental plate was assayed for necrosis using a Non-Radioactive Cell Proliferation kit (MTT Assay). Untrtd = Untreated; PIC = Poly I:C (1μg/mL); Asp1 = Aspirin (1mM); Asp5 = Aspirin (5mM); DHA = Docosahexaenoic Acid (50μM). * = p<0.05; ** = p<0.005; *** = p<0.0005. **C**. 50,000 EOC20 Microglia were plated in a 96 well plate containing Complete Medium plus 20% LADMAC Conditioned Medium. Cells were stimulated with 50μM DHA for 24 hours. After 24 hours, cells were stimulated with Imiquimod, Aspirin at 1mM, Aspirin at 5mM, or a combination of Imiquimod and Aspirin. At 24 hours post Imiquimod stimulation and/or Aspirin treatment, the experimental plate was assayed for necrosis using a Non-Radioactive Cell Proliferation kit (MTT Assay). Untrtd = Untreated; Imi = Imiquimod (10μg/mL); Asp1 = Aspirin (1mM); Asp5 = Aspirin (5mM); DHA = Docosahexaenoic Acid (50μM). * = p<0.05; *** = p<0.0005; **** = p<0.00005; ***** = p<0.000005.

EOC20 microglia were pretreated with DHA prior to stimulation with TLR agonists, Aspirin treatment, or combination. The MTT assay was used to determine if DHA pretreatment, Aspirin treatment, or a combination of both were toxic to EOC20 microglia. Absorbance values of each treatment were compared to the untreated group. Any treatment that had an absorbance below the average absorbance for the untreated was considered as toxic. Any treatment that had an absorbance above the average absorbance for the untreated was deemed non-toxic. DHA pretreatment did not cause toxicity (Figure 1A). Treatments with Aspirin at either 1mM or 5mM were also non-toxic (Figure 1A). DHA pretreatment followed by 1mM Aspirin treatment was not toxic (Figure 1A). However, DHA pretreatment and 5mM Aspirin treatment was slightly toxic (Figure 1A). This toxicity was seen at 24 hours post Aspirin treatment and causes some worry as to the amount of toxicity that would be seen at 48 hours post Aspirin treatment. For this reason, 1mM was chosen as the concentration of Aspirin to be used in future experiments.

Activation by Poly I:C (TLR-3) or Imiquimod (TLR-7) were both non-toxic to EOC20 microglia at 24 hours (Figure 1B-C). DHA pretreatment prior to TLR activation was not toxic to EOC20 microglia (Figure 1B-C). Aspirin treatment at either 1mM or 5mM during TLR activation was also not toxic (Figure 1B-C). A combination of DHA pretreatment and Aspirin treatment (1mM or 5mM) was not toxic for either TLR-3 or TLR-7 activated microglia (Figure 1B-C).

### DHA pretreatment increases total Glutathione in activated microglia

**Figure 2:**
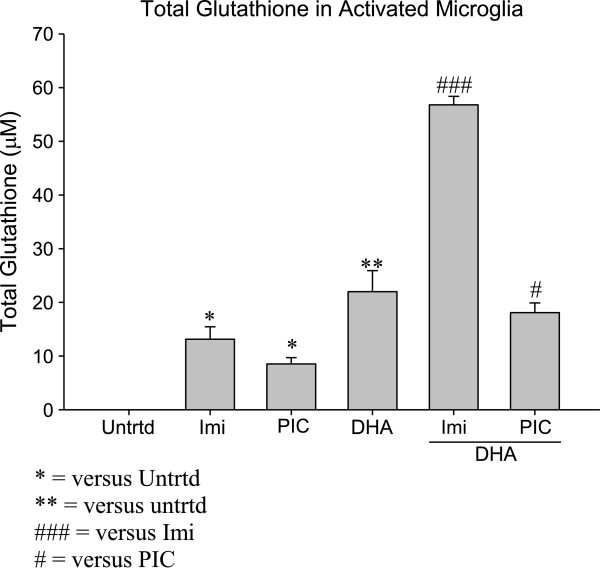
**50,000 EOC20 Microglia were plated in a 96 well plate containing Complete Medium plus 20% LADMAC Conditioned Medium.** Cells were stimulated with 50μM DHA for 24 hours. After 24 hours, cells were stimulated with TLR agonists. At 24 hours post TLR stimulation, supernatants and cellular extracts were collected; total incubation time was 48 hours. Cellular extracts were assayed for total Glutathione content. Values were normalized to the untreated. Untrtd = Untreated; Imi = Imiquimod (10μg/mL); PIC = Poly I:C (1μg/mL); DHA = Docosahexaenoic Acid (50μM). *,# = p< 0.05; ** = p<0.005, ### = p<0.0005.

Microglia were activated with TLR agonists after a 24 hour pretreatment with DHA. The values were normalized to the untreated. Results show that Poly I:C and Imiquimod both cause a modest increase in total Glutathione (8.5μM and 13μM, respectively; Figure 2). These increases in Glutathione are significant (p < 0.05) and indicate that both Poly I:C (TLR-3) and Imiquimod (TLR-7) agonists can activate EOC20 microglia and induce a Glutathione-based anti-oxidative mechanism.

When compared to TLR-3 and TLR-7 stimulations where no DHA was present, DHA pretreatment significantly increased total Glutathione (18.1μM and 56.8μM, respectively) in both simulated viral infection environments (p < 0.05 and p < 0.0005, respectively; Figure 2). This confirms that DHA can increase total Glutathione and enhance the anti-oxidative capacity of activated EOC20 microglia.

### DHA pretreatment plus aspirin increase total Glutathione in activated microglia

**Figure 3:**
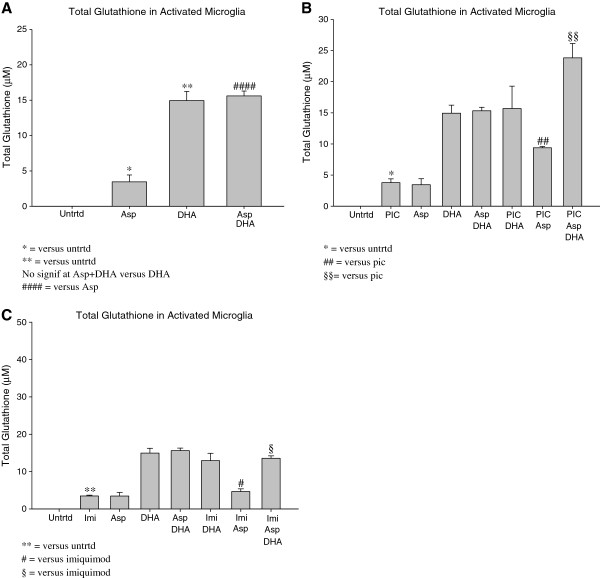
**A. 50,000 EOC20 Microglia were plated in a 96 well plate containing Complete Medium plus 20% LADMAC Conditioned Medium.** Cells were stimulated with 50μM DHA for 24 hours. After 24 hours, cells were treated with 1mM Aspirin. At 24 hours post Aspirin treatment, supernatants and cellular extracts were collected; total incubation time was 48 hours. Cellular extracts were assayed for total Glutathione content. Values were normalized to the untreated. Untrtd = Untreated; Asp = Aspirin (1mM); DHA = Docosahexaenoic Acid (50μM). * = p< 0.05; ** = p<0.005, #### = p<0.00005. **3B.** 50,000 EOC20 Microglia were plated in a 96 well plate containing Complete Medium plus 20% LADMAC Conditioned Medium. Cells were stimulated with 50μM DHA for 24 hours. After 24 hours, cells were treated with Poly I:C, 1mM Aspirin, or a combination of Poly I:C and Aspirin. At 24 hours post Poly I:C and/or Aspirin treatment, supernatants and cellular extracts were collected; total incubation time was 48 hours. Cellular extracts were assayed for total Glutathione content. Values were normalized to the untreated. Untrtd = Untreated; PIC = Poly I:C (1μg/mL); Asp = Aspirin (1mM); DHA = Docosahexaenoic Acid (50μM). * = p<0.05; **,##,§§ = p<0.005. **3C.** 50,000 EOC20 Microglia were plated in a 96 well plate containing Complete Medium plus 20% LADMAC Conditioned Medium. Cells were stimulated with 50μM DHA for 24 hours. After 24 hours, cells were treated with Imiquimod, 1mM Aspirin, or a combination of Imiquimod and Aspirin. At 24 hours post Imiquimod and/or Aspirin treatment, supernatants and cellular extracts were collected; total incubation time was 48 hours. Cellular extracts were assayed for total Glutathione content. Values were normalized to the untreated. Untrtd = Untreated; Imi = Imiquimod (10μg/mL); Asp = Aspirin (1mM); DHA = Docosahexaenoic Acid (50μM). *,#,§ = p<0.05; ** = p<0.005.

EOC20 microglia were pretreated with DHA prior to TLR stimulation, Aspirin treatment, or both. DHA pretreatment, alone, increased total Glutathione production to about 15μM (p < 0.005; Figure 3A). Aspirin (1mM) treatment increased total Glutathione production to 3.5μM (p < 0.05; Figure 3A). DHA pretreatment prior to Aspirin (1mM) treatment increased total Glutathione production to 15.6μM (Figure 3A). This increase in total Glutathione is significant (p < 0.00005) when compared to total Glutathione produced by 1mM Aspirin alone. This suggests that both DHA and Aspirin are effective in activating a Glutathione-based anti-oxidative mechanism in EOC20 microglia.

Poly I:C induced TLR-3 activation increased total Glutathione production to 3.7μM (p < 0.05; Figure 3B). Treatment with 1mM Aspirin during TLR-3 activation increased total Glutathione production to 9.4μM (p < 0.005; Figure 3B). DHA pretreatment prior to TLR-3 activation and Aspirin (1mM) treatment further increased total Glutathione production to 23.8μM (p < 0.005; Figure 3B). The augmentation of total Glutathione in the presence of DHA plus Aspirin and Poly I:C is noteworthy. This synergism was not observed in the case of DHA, Aspirin and Imiquimod.

Microglia activated by Imiquimod, a TLR-7 agonist, produced 3.5μM total Glutathione (p < 0.005; Figure 3C). Treatment with 1mM Aspirin during TLR-7 activation increased total Glutathione production to 4.6μM (p < 0.1; Figure 3C). Total Glutathione production was further increased when EOC20 were pretreated with DHA prior to TLR-7 activation and Aspirin (1mM) treatment (13.6μM; p < 0.05; Figure 3C).

### DHA pretreatment reduces Pro-inflammatory cytokine secretion by activated microglia

**Figure 4:**
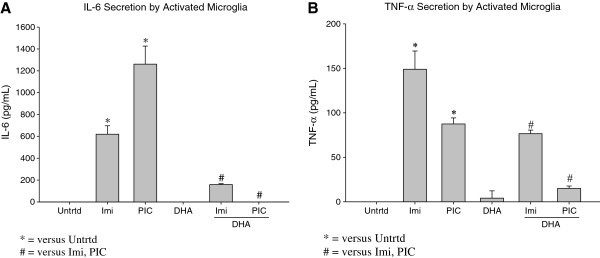
**A. 50,000 EOC20 Microglia were plated in a 96 well plate containing Complete Medium plus 20% LADMAC Conditioned Medium.** Cells were stimulated with 50μM DHA for 24 hours. After 24 hours, cells were stimulated with TLR agonists. At 24 hours post TLR stimulation, supernatants were collected; total incubation time was 48 hours. IL-6 secretion was determined by assaying the supernatants with an IL-6 ELISA. Values were normalized to the untreated. Untrtd = Untreated; Imi = Imiquimod (10μg/mL); PIC = Poly I:C (1μg/mL); DHA = Docosahexaenoic Acid (50μM). *,# = p< 0.05. **4B.** 50,000 EOC20 Microglia were plated in a 96 well plate containing Complete Medium plus 20% LADMAC Conditioned Medium. Cells were stimulated with 50μM DHA for 24 hours. After 24 hours, cells were stimulated with TLR agonists. At 24 hours post TLR stimulation, supernatants were collected; total incubation time was 48 hours. TNF- α secretion was determined by assaying the supernatants with a TNF- α ELISA. Values were normalized to the untreated. Untrtd = Untreated; Imi = Imiquimod (10μg/mL); PIC = Poly I:C (1μg/mL); DHA = Docosahexaenoic Acid (50μM). *,# = p< 0.05.

EOC20 microglia were pretreated with DHA for 24 hours, and then stimulated with a TLR agonist. IL-6 secretion was measured using an IL-6 ELISA assay, and values were normalized to the untreated. Results show that DHA pretreatment does not induce baseline IL-6 production (Figure 4A). Activation with Poly I:C or Imiquimod both significantly increased IL-6 production (p < 0.05, Figure 4A). Poly I:C caused secretion of approximately 1200pg/mL of IL-6, whereas Imiquimod resulted in approximately 600pg/mL (Figure 4A). This indicates that Poly I:C (TLR-3 agonist) stimulation causes a much more robust IL-6 response than Imiquimod.

When pre-treated with DHA prior to TLR stimulation, a significant reduction on IL-6 secretion is seen. DHA caused IL-6 secretion to be undetectable in EOC20 microglia activated by Poly I:C (p < 0.05, Figure 4A). This indicates that DHA can effectively inhibit IL-6 pro-inflammatory cytokine production in microglia activated by a TLR-3 agonist (Poly I:C). DHA pretreatment prior to Imiquimod (TLR-7) activation caused IL-6 secretion to be significantly reduced to 159pg/mL (p < 0.05; Figure 4A). This indicates that DHA can also modulate IL-6 pro-inflammatory cytokine production in EOC20 microglia activated by a TLR-7 agonist.

EOC20 microglia were stimulated with a TLR-3 or TLR-7 agonist after a 24 hour pretreatment with DHA. TNF-α secretion was measured using a TNF-α ELISA, and values were normalized to the untreated. Pretreatment with DHA caused a low butd insignificant production of baseline TNF-α (Figure 4B). Poly I:C and Imiquimod both significantly increased TNF-α secretion (80pg/mL and 150pg/mL, respectively; p < 0.05; Figure 4B). This data shows that EOC20 microglial activation with Poly I:C (TLR-3) or Imiquimod (TLR-7) yields a pro-inflammatory response that involves TNF-α, in addition to IL-6.

When pre-treated with DHA for 24 hours prior to TLR stimulation, TNF-α secretion was significantly reduced (Figure 4B). DHA reduced TNF-α secretion by Poly I:C activated microglia to 15pg/mL (p < 0.05; Figure 4B). DHA pretreatment prior to Imiquimod activation reduced TNF-α secretion to 76.5pg/mL (p < 0.05; Figure 4B). This data suggests that DHA can decrease TNF-α pro-inflammatory cytokine secretion by EOC20 microglia that are activated by a TLR-3 or TLR-7 agonist.

Both sets of data indicate that DHA can effectively exert its anti-inflammatory properties on microglia that are activated by either a TLR-3 or TLR-7 agonist (Poly I:C or Imiquimod, respectively).

### DHA pretreatment plus Aspirin cause Pro-inflammatory cytokine production to be markedly reduced in activated microglia

**Figure 5:**
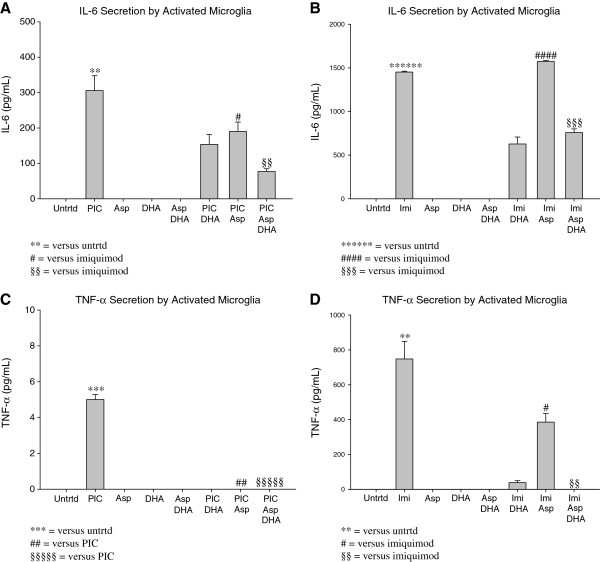
**A. 50,000 EOC20 Microglia were plated in a 96 well plate containing Complete Medium plus 20% LADMAC Conditioned Medium.** Cells were stimulated with 50μM DHA for 24 hours. After 24 hours, cells were stimulated with Poly I:C, 1mM Aspirin, or a combination of Poly I:C and Aspirin. At 24 hours post Poly I:C and/or Aspirin treatment, supernatants were collected; total incubation time was 48 hours. IL-6 ELISAs determined IL-6 secretion. Values were normalized to the untreated. Untrtd = Untreated; PIC = Poly I:C (1μg/mL); Asp = Aspirin (1mM); DHA = Docosahexaenoic Acid (50μM). # = p< 0.05; **,§§ = p<0.005. **5B**. 50,000 EOC20 Microglia were plated in a 96 well plate containing Complete Medium plus 20% LADMAC Conditioned Medium. Cells were stimulated with 50μM DHA for 24 hours. After 24 hours, cells were stimulated with Imiquimod, 1mM Aspirin, or a combination of Imiquimod and Aspirin. At 24 hours post Imiquimod and/or Aspirin treatment, supernatants were collected; total incubation time was 48 hours. IL-6 ELISAs determined IL-6 secretion. Values were normalized to the untreated. Untrtd = Untreated; Imi = Imiquimod (10μg/mL); Asp = Aspirin (1mM); DHA = Docosahexaenoic Acid (50μM). §§§ = p<0.0005; #### = p<0.00005; ****** = p<0.0000005. **5C**. 50,000 EOC20 Microglia were plated and stimulated as in 5A. TNF- α ELISAs determined TNF- α secretion. Values were normalized to the untreated. Untrtd = Untreated; PIC = Poly I:C (1μg/mL); Asp = Aspirin (1mM); DHA = Docosahexaenoic Acid (50μM). ## = p< 0.005; *** = p< 0.0005; §§§§§ = p<0.000005. **5D**. 50,000 EOC20 Microglia were plated and stimulated as in 5B. TNF- α ELISAs determined TNF- α secretion. Values were normalized to the untreated. Untrtd = Untreated; Imi = Imiquimod (10μg/mL); Asp = Aspirin (1mM)DHA = Docosahexaenoic Acid (50μM). # = p<0.05; **, §§ = p<0.005.

EOC20 microglia were pretreated with DHA prior to TLR stimulation, Aspirin treatment, or both. IL-6 pro-inflammatory cytokine production was measured using an IL-6 ELISA; values were normalized to the untreated. Figures 5A-B show that Aspirin (1mM) treatment doesn’t induce IL-6 production, as expected. DHA pretreatment prior to Aspirin (1mM) treatment also doesn’t induce IL-6 production (Figure 5A-B). This indicates that neither Aspirin treatment, nor DHA pretreatment prior to TLR stimulation, affect baseline IL-6 production.

TLR-3 activation by Poly I:C significantly increased IL-6 production to 305.9 pg/mL (p < 0.005; Figure 5A). When treated with Aspirin (1mM) during TLR-3 activation, IL-6 secretion was reduced to 190.3pg/mL (p < 0.05; Figure 5A). DHA pretreatment prior to TLR-3 activation and Aspirin treatment further reduced IL-6 secretion by activated microglia (77.11pg/mL; p < 0.005; Figure 5A).

EOC20 microglia activated by Imiquimod (TLR-7) secreted 1451.8pg/mL IL-6 (p < 0.0000006; Figure 5B). When treated with Aspirin during TLR-7 activation, IL-6 production was slightly increased (1574.14pg/mL; Figure 5B). Aspirin’s inability to reduce production of IL-6 when the stimulus is a TLR-7 agonist (Imiquimod) is rather surprising. Pretreatment with DHA prior to TLR-7 activation and Aspirin treatment significantly reduces IL-6 production to 757.9pg/mL (p < 0.0005; Figure 5B).

These results indicate that the presence of DHA prior to TLR stimulation is very important when trying to reduce IL-6 pro-inflammatory cytokine production in response to TLR-3 or TLR-7 activation.

TNF-α pro-inflammatory cytokine production was measured with a TNF-α ELISA, and values were normalized to the untreated. Figures 5C-D indicate that Aspirin (1mM) treatment has no effect on TNF-α production. TNF-α is also undetectable in EOC20 microglia cultures that were pretreated with DHA prior to Aspirin (1mM) treatment (Figure 5C-D). These results show that neither Aspirin treatment, nor DHA pretreatment prior to Aspirin treatment, affect baseline TNF-α production.

Poly I:C (TLR-3) stimulation caused a modest but statistically significant production of TNF-α (5.0pg/mL; p < 0.0005; Figure 5C). Treatment with 1mM Aspirin during TLR-3 activation caused TNF-α to be undetectable (p < 0.005; Figure 5C). This indicates that Aspirin exerts anti-inflammatory properties during a TLR-3 simulated infectious environment. Pretreatment with DHA prior to TLR-3 activation and Aspirin treatment also caused TNF-α production to be undetectable (p < 0.000005; Figure 5C).

TLR-7 activation by Imiquimod caused 747.8pg/mL of TNF-α to be secreted by EOC20 microglia (p < 0.005; Figure 5D). Treatment with 1mM Aspirin during TLR-7 activation significantly reduced TNF-α production to 385.9pg/mL (p < 0.05; Figure 5D). Pretreatment with DHA prior to TLR-7 activation and Aspirin treatment caused TNF-α production to be undetectable (p < 0.005; Figure 5D).

When taken together, the results of the IL-6 and TNF-α ELISAs suggest that DHA pre-treatment prior to TLR activation is the most effective way to reduce pro-inflammatory cytokine production. Furthermore, Aspirin treatment can enhance the reduction in pro-inflammatory cytokines with the notable exception of Imiquimod and IL-6.

### DHA pretreatment reduces total Nitric Oxide secreted by activated microglia

Poly I:C and Imiquimod cause a significant increase in Nitric Oxide in EOC20 microglia cells (15μM and 16.7μM, respectively; p < 0.005). This indicates that both TLR-3 and TLR-7 activation of EOC20 microglia can cause an inflammatory response and oxidative stress that is partially mediated by Nitric Oxide.

**Figure 6:**
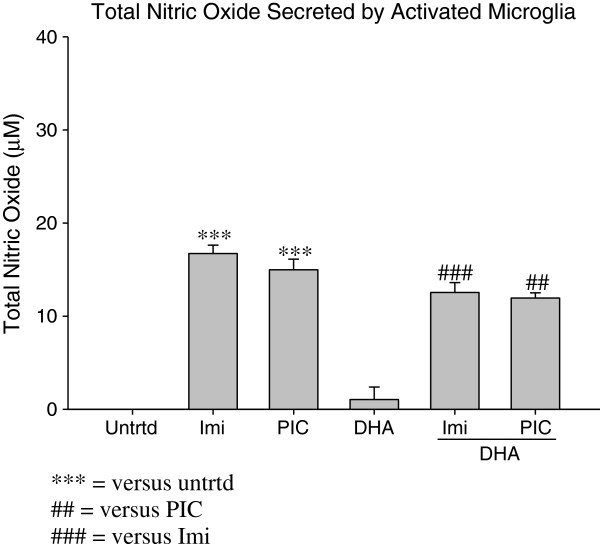
**50,000 EOC20 Microglia were plated in a 96 well plate containing Complete Medium plus 20% LADMAC Conditioned Medium.** Cells were stimulated with 50μM DHA for 24 hours. After 24 hours, cells were stimulated with TLR agonists. At 24 hours post TLR stimulation, supernatants were collected; total incubation time was 48 hours. Supernatants were assayed for total Nitric Oxide content using a total Nitric Oxide kit. Values were normalized to the untreated. Untrtd = Untreated; Imi = Imiquimod (10μg/mL); PIC = Poly I:C (1μg/mL); DHA = Docosahexaenoic Acid (50μM). ## = p<0.005; ***,### = p<0.0005.

DHA pretreatment prior to TLR-3 or TLR-7 activation caused a significant decrease in Total Nitric Oxide production. DHA pretreatment prior to Poly I:C stimulation reduced total Nitric Oxide production to 11.95μM (p < 0.005; Figure 6). Total Nitric Oxide was reduced to 12.74 μM when microglia were pretreated with DHA prior to Imiquimod stimulation (p < 0.0005; Figure 6). This suggests that DHA can reduce Nitric Oxide-based inflammation and oxidative stress that occur in EOC20 microglia when activated by a TLR-3 or TLR-7 agonist.

### DHA pretreatment plus Aspirin cause total Nitric Oxide to be undetectable in activated microglia

**Figure 7:**
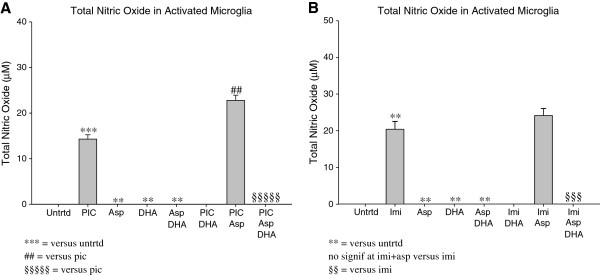
**A. 50,000 EOC20 Microglia were plated in a 96 well plate containing Complete Medium plus 20% LADMAC Conditioned Medium.** Cells were stimulated with 50μΜ aspirin or a combination of aspirin and Poly I:C. At 24 hours post Poly I:C and/or asprin treatment, supernatants were collected; total incubation time was 48 hrs. Supernatants were assayed for total Nitric Oxide using a total Nitric Oxide kit. Values were normalized to the untreated. Untrtrd=Untreated; PIC=Poly I:C (1μg/mL); Asp = Aspirin (1mM); DHA = Docosahexaenoic Acid (50μM). **, ## = p<0.005; *** = p<0.0005; §§§§§ = p<0.00005. **B.** 50,000 EOC20 Microglia were plated in a 96 well plate containing Complete Medium plus 20% LADMAC Conditioned Medium. Cells were stimulated with 50μM DHA for 24 hours. After 24 hours, cells were stimulated with Imiquimod, 1mM Aspirin, or a combination of Imiquimod and Aspirin. At 24 hours post Imiquimod and/or Aspirin treatment, supernatants were collected; total incubation time was 48 hours. Supernatants were assayed for total Nitric Oxide content using a total Nitric Oxide kit. Values were normalized to the untreated. Untrtd = Untreated; Imi = Imiquimod (10μg/mL); Asp = Aspirin (1mM); DHA = Docosahexaenoic Acid (50μM). ** = p<0.005; §§§ = p<0.0005.

Pretreatment with DHA for 24 hours caused total Nitric Oxide to be undetectable, indicating that DHA does not induce baseline Nitric Oxide (p < 0.005; Figures 7A-B). This result is also seen when EOC20 microglia are treated with 1mM Aspirin alone (p < 0.005; Figures 7A-B). Total Nitric Oxide production was also undetectable in EOC20 microglia that were pretreated with DHA prior to Aspirin (1mM) treatment (p < 0.005; Figures 7A-B). This indicates that the combined therapy of DHA pretreatment and Aspirin treatment does not influence baseline Nitric Oxide production.

Microglia activated by Poly I:C (TLR-3) produced 14.3μM total Nitric Oxide (p < 0.0005; Figure 7A). Treatment with Aspirin (1mM) increased total Nitric Oxide production by Poly I:C activated microglia (22.78μM; Figure 7A). This increase in total Nitric Oxide was significant (p < 0.005), indicating that 1mM Aspirin could be augmenting Nitric Oxide production in TLR-3 activated microglia, rather than reducing it. Total Nitric Oxide was undetectable in TLR-3 activated microglia that were pretreated with DHA prior to TLR-3 activation and Aspirin treatment (p < 0.000005; Figure 7A).

TLR-7 activation by Imiquimod increased total Nitric Oxide secretion to about 20μM (p < 0.005; Figure 7B). Aspirin (1mM) increased total Nitric Oxide secretion by TLR-7 activated microglia (24.1μM; Figure 7B). However, this modest increase in total Nitric Oxide due to Aspirin (1mM) treatment was not significant (p>0.1). Total Nitric Oxide was undetectable in TLR-7 activated microglia that were pretreated with DHA prior to TLR activation and Aspirin treatment (p < 0.0005; Figure 7B).

## Conclusions

Epidemiological studies show an inverse relationship between NSAID intake and the development of Alzheimer’s and Parkinson’s diseases [23,27]. NSAIDs that specifically target COX-2 have been shown to reduce microglia activation, block the production of pro-inflammatory cytokines, and reduce the risk of Alzheimer’s disease [4]. NSAIDs are also proposed to have an effect on the inflammatory component of Multiple Sclerosis and Amyotrophic Lateral Sclerosis [27]). DHA plays a key role in proper brain development and in maintaining a homeostatic environment in the CNS [15,18,19]. DHA has been shown to modulate important neurochemical processes, synaptic plasticity, memory formation, neuroprotection, gene expression, and intracellular calcium concentrations [15,19]. DHA has also been shown to promote neuronal stem cell differentiation into neurons, as well as neurogenesis [15]. Increased dietary intake has been shown to significantly alter DHA levels in the brain, suggesting that DHA could be used as a way to directly influence brain function [17]. DHA has potent anti-inflammatory properties in both acute and chronic neurodegenerative diseases [15]. DHA has been shown to reduce the number of activated microglia and reduce pro-inflammatory molecule production [15]. Clinical studies suggest dietary DHA supplementation can alter the risk of developing Alzheimer’s disease [17].

Our findings indicate that DHA pretreatment enhances total Glutathione production in activated microglia. This suggests that DHA has the ability to increase the anti-oxidative capacity of EOC20 microglia, allowing the cells to combat oxidative stress that occurs as a result of activation. DHA pretreatment also significantly reduced IL-6 and TNF-α pro-inflammatory cytokine production during both TLR-3 and TLR-7 microglia activation, proving that DHA can exert its anti-inflammatory properties in both types of simulated viral infections. Total Nitric Oxide was also reduced when EOC20 were pretreated with DHA prior to TLR activation. This suggests that DHA can reduce NO-based inflammation and oxidative stress that occur in EOC20 microglia when activated by a TLR-3 or TLR-7 agonist.

Aspirin treatment alone enhanced baseline tGSH production, indicating that Aspirin can also influence the anti-oxidative capacity of EOC20 microglia in similar manner to DHA. When combined with DHA pretreatment, a further increase in total Glutathione production was observed. This indicates that DHA pretreatment prior to Aspirin treatment may be necessary in order to further enhance the Glutathione-based anti-oxidative mechanism that is activated by Aspirin.

IL-6 production as a result of TLR-3 activation was reduced when treated with Aspirin. This indicates that Aspirin exerts its anti-inflammatory properties on TLR-3 activated EOC20 microglia. TNF-α production was undetectable when treated with Aspirin during TLR-3 activation, indicating that Aspirin affects TNF-α production much more than it affects IL-6 production. Aspirin treatment during TLR-7 activation slightly increased IL-6 production. This indicates that concurrent Aspirin treatment and TLR-7 activation is rather ineffective in modulating IL-6 production arising from a TLR-7 stimulus. However, Aspirin treatment did significantly reduce TNF-α production by TLR-7 activated microglia. This causes us to believe that Aspirin does, in fact, affect TNF-α production much more than it affects IL-6 production. DHA pretreatment prior to either TLR-3 or TLR-7 activation and Aspirin treatment significantly reduced IL-6 production, indicating that DHA pretreatment is the most effective way to reduce pro-inflammatory cytokine production in activated microglia.

Aspirin treatment during TLR-3 activation caused a significant increase in total Nitric Oxide. This raises the possibility that use of Aspirin as an anti-inflammatory molecule during TLR-3 simulated viral infection is ineffective. An increase in total Nitric Oxide was also seen when treated with Aspirin during TLR-7 induced microglia activation. This increase in total Nitric Oxide was not significant, therefore we do not suspect Aspirin as being problematic during TLR-7 induced microglia activation. When pretreated with DHA prior to TLR activation and Aspirin treatment, total Nitric Oxide was undetectable, indicating that DHA is very important for reducing total Nitric Oxide production.

Collectively, these findings highlight the combination of DHA and Aspirin as a possible preventative measure against neurodegenerative diseases. Animal model studies would prove valuable in determining the overall effect of DHA plus Aspirin dietary supplements as a means to prevent/delay the onset of neurodegenerative diseases.

## Methods

### Preparation of LADMAC conditioned medium

LADMAC cells were purchased from the American Type Culture Collection (ATCC; Manassas, VA) and used to produce conditioned medium. Briefly, LADMAC were cultured in 175cm^2^ tissue culture flasks containing complete medium comprised of 10% Fetal Bovine Serum (FBS), 1% Antibiotic/Antimycotic (Penicillin-Streptomycin/Amphotericin B), 0.5% Gentamycin, and Dulbecco’s Modified Eagle Medium. Cells were grown to 100% confluency and supernatants were transferred to a 50mL tube and spun at 3000xg for 15 minutes. The supernatants were then passed through a 0.22 micron filter to remove any remaining cells. Supernatants were then stored at −20°C for future use.

### Preparation of EOC20 microglia cultures

EOC20 microglia were obtained from the ATCC (Manassas, VA). EOC20 were cultured in a 25cm^2^ tissue culture flask containing complete medium consisting of 10% Fetal Bovine Serum (FBS), 20% LADMAC Conditioned Medium (as described above), 1% Antibiotic/Antimycotic (Penicillin-Streptomycin/Amphotericin B), 0.5% Gentamycin, and Dulbecco’s Modified Eagle Medium. Upon 100% confluency, EOC20 were cultured in 96 well plates at 200,000cells/mL and 250μL/well, giving a final cell concentration of 50,000cells/well. Two separate experimental set ups were used.

Experimental Setup #1: Cells were pretreated with 50μM Docosahexaenoic Acid (DHA; Cayman Chemicals; Ann Arbor, MI) for 24 hours. At 24 hours post DHA pretreatment, EOC20 microglia were stimulated with either 1μg/mL Polyinosinic-Polycytidylic acid (Poly I:C; InvivoGen; San Diego, CA), a TLR-3 agonist that mimics viral double stranded RNA, or 10μg/mL Imiquimod (InvivoGen; San Diego, CA), a TLR-7 agonist that mimics single stranded RNA. TLR stimulation was for 24 hours, leading to a total incubation time of 48 hours. At the end of the 48 hours, supernatants were collected and stored at −20°C. The experimental plate was washed with 1x Phosphate Buffered Saline (PBS). 100μL 5% Metaphosphoric Acid (MPA) was added to each experimental well, and the plate was frozen at −80°C.

Experimental Setup #2: Cells were pretreated with 50μM Docosahexaenoic Acid (DHA; Cayman Chemicals; Ann Arbor, MI) for 24 hours. At 24 hours post DHA pretreatment, EOC20 microglia were stimulated with either 1μg/mL Polyinosinic-Polycytidylic acid (Poly I:C; InvivoGen; San Diego, CA), 10μg/mL Imiquimod (InvivoGen; San Diego, CA), 1mM Aspirin (Sigma-Aldrich Chemicals; Saint Louis, MO), 5mM Aspirin, or a combination of TLR agonist and one of the aforementioned concentrations of Aspirin. TLR and/or Aspirin stimulation was for 24 hours, leading to a total incubation time of 48 hours. At the end of the 48 hours, supernatants were collected and stored at −20°C. The experimental plate was washed with 1x Phosphate Buffered Saline (PBS). 100μL 5% Metaphorphoric Acid (MPA) was added to each experimental well, and the plate was frozen at −80°C.

### MTT cell proliferation

Treatment toxicity was determined using a Non-Radioactive Cell Proliferation Assay (Promega; Madison, WI), following the manufacturer’s instructions. Briefly, an experimental plate was set up following the guidelines outlined under ‘Experimental Setup #2’. Cells in PBS were also plated as a positive control for necrosis. At 24 hours post TLR and/or Aspirin stimulation, the MTT cell proliferation assay was performed. 100μL of supernatant was removed from the experimental plate, leaving 150μL of supernatant in each well. Dye solution (15μL) containing tetrazolium was added to each well and the plate was incubated at 37°C, 10% CO_2_ for 4 hours. During this incubation, tetrazolium salt in the dye solution is converted to a formazan product by living cells. After incubation, 100μL solubilization/stop Solution was added to each well in order to solubilize the formazan present in each well. The plate was incubated for 1.5 hours at room temperature. Absorbance values were determined at 570nm. Necrosis was determined by comparing the treated wells to the untreated wells. The student t-test was used to determine significance (p-values).

### Total Glutathione production assay

Total Glutathione (tGSH) in activated EOC20 microglia cells was measured colorimetrically using a Total Glutathione (tGSH) Microplate Assay kit (Eagle Biosciences; Boston, MA). Briefly, the experimental plate was washed with 1x Phosphate Buffered Saline (PBS) after supernatants were collected. 100uL 5% Metaphosphoric Acid (MPA) was added to each experimental well, and the plate was frozen at −80°C. The experiment plates were then thawed at 37°C. After the second freeze-thaw cycle, the plate was put on ice for 15 minutes. After 15 minutes, 50μL of MPA extract was removed from each well and placed into an assay plate. A total Glutathione standard curve was made following the manufacturer’s instructions and added to the assay plate. Next, 5,5’-dithiobis(2-nitrobenzoic acid) [DTNB], Glutathione oxidoreductase, and beta-Nicotinamide Adenine Dinucleotide Phosphate (β-NADPH) were added to each well, allowing Glutathione to reduce DTNB and form a mixed disulfide and a colored ion. This mixed disulfide then reacted with Glutathione that is present in the sample to form GSSG and another colored ion. GSSG re-enters this cycle once it is reduced enzymatically. The experimental plate was read at 405nm at 0 minutes and 10 minutes after the addition of β-NADPH. Absorbance values were then used to determine the concentration of total Glutathione (GSH and GSSG) that is present in the sample. The sample values were normalized to the untreated sample. Significance was determined using the student t-test.

### Pro-inflammatory cytokine assays

IL-6 and TNF-α secretion was determined using IL-6 and TNF-α Enzyme Linked Immunosorbent Assays (ELISAs) (BD Falcon; San Diego, CA). Briefly, ELISA plates were coated with IL-6 or TNF-α capture antibody and incubated at 4°C for 24 hours. The final concentration of the capture antibody was 1:250. After 24 hours, the plate was washed with wash buffer that was made following the manufacturer’s instructions. The plate was then blocked for 1 hour at room temperature using 200μL assay diluent (10% FBS in PBS). Supernatants collected from experimental setup #1 and #2 were thawed and diluted 1:2. The diluted samples and an IL-6 or TNF-α standard curve were added to the plate after the one hour blocking step and another round of washing. Sample and standard curve incubation was at room temperature and lasted 2 hours. After another round of washing, IL-6 or TNF-α Detection antibody (1:250) and Streptavidin-Horse Radish Peroxidase Enzyme Reagent (1:250) were added to each well and incubated for one hour at room temperature. After incubating, the ELISA plates were washed seven times using a plate shaker. 100μL TMB substrate solution containing Hydrogen Peroxide and Tetramethylbenzidine (1:1) was added to each well. The plate was incubated for 30 minutes in the dark followed by an addition of 50 μL 2N sulfuric acid and read at 450nm. Absorbance values were then used to determine IL-6 and TNF-α secretion using the appropriate standard curve. Significance was determined using the student t-test.

### Total Nitric Oxide secretion assay

Total Nitric Oxide Secretion was determined using a Total Nitric Oxide Assay Kit (Thermo Scientific; Rockford, IL), following the manufacturer’s instructions. Briefly, supernatants collected from experimental setup #1 and #2 were thawed and added to assay plates. A Nitrate standard curve was also added to the experimental plate. Nicotinamide Adenine Dinucleotide Phosphate (NADPH) and Nitrate Reductase were added to each sample and standard. The plate was then incubated at 37°C for 30 minutes, allowing Nitrate Reductase to function optimally and convert any Nitrate into Nitrite. After the 30 minute incubation, Greiss Reagents I and II were added to each sample. The plate was then incubated for 10 minutes at room temperature, allowing the Greiss Reagents to react with Nitrite and produce a colored azo dye product. The plate was read at 540nm and absorbance values for each sample were recorded. The average absorbance of the blank was subtracted from the absorbance values for each sample. The samples were then normalized to the untreated sample. Significance was determined using the student t-test, p < 0.05 were considered statistically significant.

## Abbreviations

DHA: Docosahexaenoic Acid; PUFA: Poly Unsaturated Fatty Acid; NSAIDs: Non Steroidal Anti-Inflammatory; COX: Cyclooxygenase; tGSH: total Glutathione; TLR: Toll Like Receptors; IL: Interleukin; TNF-α: Tumor Necrosis Factor-alpha; NO: Nitric Oxide; ROS: Reactive Oxygen Species; Poly I:C: Polyinosinic:polycytidylic.

## Competing interests

The authors declare that they have no competing interests.

## Authors’ contributions

LP carried out the majority of the experiments and manuscript preparation. LP and EV had substantial contributions to conception, design, interpretation of data. CV and JT carried out culturing and some experiments. All authors read and approved the final manuscript.
